# P-1532. Surveillance of Eravacycline Against Clinical Pathogens, Including Resistant Isolates, Collected Worldwide from Multiple Infection Sites During 2018-2022

**DOI:** 10.1093/ofid/ofae631.1700

**Published:** 2025-01-29

**Authors:** Stephen Hawser, Federica Monti, Sherry Siegert, Kristie Zappas, Sarah McLeod, Samir H Moussa

**Affiliations:** IHMA Europe, Monthey, Valais, Switzerland; IHMA, Monthey, Valais, Switzerland; Innoviva Specialty Therapeutics, Inc., Waltham, Massachusetts; Innoviva Specialty Therapeutics, Inc., Waltham, Massachusetts; Innoviva Specialty Therapeutics, Inc., Waltham, Massachusetts; Innoviva Specialty Therapeutics, Inc., Waltham, Massachusetts

## Abstract

**Background:**

Eravacycline is a fully synthetic, fluorocycline antibiotic approved for the treatment of complicated intra-abdominal infections (cIAI) in patients ≥18 years of age in Europe, Singapore, China, Taiwan, Hong Kong, the US and the UK. The purpose of this study was to monitor the *in vitro* activity of eravacycline against isolates collected from a global surveillance study from 2018-2022. A total of 35,336 isolates were collected of which data for 23,127 isolates comprising *Staphylococcus aureus* (including methicillin-resistant *S. aureus*, MRSA), *Enterococcus* spp. (including vancomycin-resistant *Enterococcus*, VRE) and *Streptococcus* spp., *Enterobacterales* (*C. freundii*, *E. cloacae, E. coli*, *K. oxytoca* and *K. pneumoniae*) and non-fermenters are reported.

**Figure:**
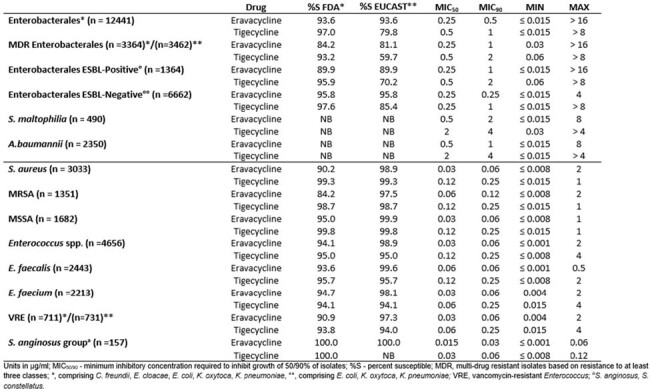

**Methods:**

A total of 23,127 clinical isolates were collected during 2018-2022 from multiple infection sources, including, IAI, body fluids, blood, gastrointestinal, genitourinary and respiratory. The isolates were from Asia/Pacific (n=4,812), Europe (n=9,606) and North America (n=8,709). Minimum inhibitory concentrations (MICs) were determined by CLSI broth microdilution. For comparative purposes, isolate susceptibility for eravacycline and tigecycline was determined with both FDA and EUCAST breakpoints, where available.

**Results:**

Summary MIC data for eravacycline and tigecycline as a selected comparator are shown in the Table. Susceptibility to eravacycline in all Gram-positive pathogens were greater than 90% for most species /phenotypes though was notably higher when EUCAST breakpoints were applied to the analysis. For Enterobacterales, eravacycline susceptibilities were consistently >93% though MDR susceptibility was slightly lower (84.2% susceptible to eravacycline).

**Conclusion:**

Eravacycline demonstrated sustained high activity rates against clinically important pathogens. Importantly, the MIC_90_ and susceptibilities did not change over time and were similar for the different geographical regions.

**Disclosures:**

**Sherry Siegert, PharmD**, Innoviva Specialty Therapeutics: Stocks/Bonds (Public Company)|Innoviva Specialty Therapeutics, Inc.: Employee **Kristie Zappas, PharmD**, Innoviva Specialty Therapeutics, Inc.: Employee **Sarah McLeod, PhD**, Innoviva Specialty Therapeutics: Employee|Innoviva Specialty Therapeutics: Stocks/Bonds (Public Company) **Samir H. Moussa, PhD**, Innoviva Specialty Therapeutics: Employee|Innoviva Specialty Therapeutics: Stocks/Bonds (Public Company)

